# Prostate Cancer Therapy via Inhibition of Negative Immune Regulation

**DOI:** 10.1155/bmri/7088370

**Published:** 2026-10-13

**Authors:** Sulagna Dutta, Pallav Sengupta, Ujjayita Chowdhury, Israel Maldonado Rosas, Jogen C. Kalita, Manesh Kumar Panner Selvam, Shubhadeep Roychoudhury

**Affiliations:** ^1^ Basic Medical Sciences Department College of Medicine Ajman University Ajman UAE ajman.ac.ae; ^2^ Center of Medical and Bio‐Allied Health Sciences Research Ajman University Ajman UAE ajman.ac.ae; ^3^ Department of Biomedical Sciences Gulf Medical University Ajman UAE gmu.ac.ae; ^4^ Department of Molecular Biology and Biotechnology Tezpur University Tezpur India tezu.ernet.in; ^5^ Citmer Reproductive Medicine Mexico City Mexico; ^6^ Department of Zoology Gauhati University Guwahati India gauhati.ac.in; ^7^ Faculty of Science Assam Down Town University Guwahati India adtu.in; ^8^ Department of Urology Tulane University School of Medicine New Orleans Louisiana USA tulane.edu; ^9^ Department of Life Science and Bioinformatics Assam University Silchar India aus.ac.in

**Keywords:** cell cycle checkpoints, cytotoxic T-lymphocyte–associated antigen 4 (CTLA-4), programmed cell death protein 1 (PD-1), prostate cancer management

## Abstract

Prostate cancer exhibits complex pathophysiology, rendering its management arduous due to heterogeneity and recalcitrance toward conventional therapeutics. The increasing focus on immune checkpoint inhibitors for treatment stems from the limited existing therapeutic approaches and the impressive effectiveness of these inhibitors demonstrated in other malignancies. Denoted as immunologically “cold” with low tumor mutation burden (TMB), prostate cancer manifests diminished immune cell infiltration in tumoral tissue, potentially culminating in inadequate response to immune checkpoint blockade. Nonetheless, a substantial proportion of metastatic castration‐resistant prostate cancer (mCRPC) patients bear germline or somatic mutations in DNA repair genes, such as BReast CAncer gene 2 (BRCA2) and ataxia telangiectasia mutated (ATM), potentially augmenting TMB and neoantigen load, thus potentially prognosticating immunotherapy responsiveness. Complete immune response activation necessitates supplementary proteins functioning as T‐cell accelerators or inhibitors, such as cytotoxic T‐lymphocyte–associated antigen 4 (CTLA‐4) and programmed cell death protein 1 (PD‐1). Immune checkpoint inhibitors were thus developed, occluding negative regulatory pathways exploited by neoplastic cells to elude the immune system. Although clinical trials appear promising, proper patient selection and surveillance are imperative due to potential immune‐related adverse events. As the field progresses, immune checkpoint inhibitors are anticipated to constitute an increasingly integral facet of the therapeutic armamentarium for prostate cancer.

## 1. Introduction

Prostate cancer is the second most commonly diagnosed malignancy among men worldwide and remains a leading cause of cancer‐related death [1]. Although localized disease is frequently curable with surgery or radiotherapy, metastatic disease remains a formidable clinical challenge. Androgen deprivation therapy has long formed the backbone of systemic treatment, yet most patients eventually progress to metastatic castration‐resistant prostate cancer, a state associated with markedly reduced survival and diminishing responsiveness to conventional agents [2]. Prostate cancer remains a refractory malignancy. Because of the lethal outcome there lies an urgent need to devise a therapeutic strategy to reduce the progression and understand the underlying mechanism [3–5].

The success of immune checkpoint blockade in melanoma and nonsmall cell lung cancer catalyzed intense interest in extending this modality to prostate cancer [6, 7]. Unlike these more immunogenic tumors, however, prostate cancer typically exhibits low tumor mutational burden (TMB), minimal neoantigen presentation, sparse cytotoxic T‐lymphocyte infiltration, and a densely immunosuppressive stroma—features that collectively classify it among the so‐called cold tumors [8]. The prostate tumor microenvironment is populated by an array of immunosuppressive cell types, including regulatory T cells (T_regs_), tumor‐associated macrophages (TAMs), cancer‐associated fibroblasts (CAFs), and myeloid‐derived suppressor cells (MDSCs) [3, 9]. These cells secrete inhibitory mediators such as interleukin‐10 (IL‐10), interleukin‐6 (IL‐6), transforming growth factor‐*β* (TGF‐*β*), vascular endothelial growth factor (VEGF), and prostaglandin E2, thereby restraining T‐cell activation and enabling immune escape [3]. Prostate cancer cells further upregulate inhibitory checkpoint ligands, including programmed death‐ligand 1 (PD‐L1), B7 ‐ homolog 3 (B7‐H3), and B7 ‐ homolog 4 (B7x), compounding this suppressive milieu [10].

Despite this immunologically unfavorable landscape, molecularly defined subgroups may respond meaningfully to immunotherapy. Tumors harboring defects in DNA damage repair genes—including Breast Cancer genes (BRCA) 1 and2, ataxia telangiectasia mutated (ATM), CD, and cyclin‐dependent kinase (CDK) 12—frequently display elevated TMB and enhanced neoantigen presentation [11]. Similarly, mismatch repair‐deficient and microsatellite instability‐high (dMMR/MSI‐H) prostate cancers, though comprising only a small minority of cases, show disproportionately favorable and sometimes durable responses to programmed cell death protein (PD)‐1 blockade [12, 13]. Prostate cancer, even though traditionally considered an “immunologically cold” tumor with poor sensitivity to immunotherapy, is heading into an entirely new world of potential treatment options based on combinatorial approaches, biomarker‐based selection of patients, and mechanism‐based novel immunotherapy options [5]. Although the conventional monotherapy approach failed to produce sustained responses to immunotherapy in early‐stage clinical trials, it is suggested that the combination of vaccines, immune checkpoint blockade, chimeric antigen receptor (CAR)‐T cells, and bispecific T‐cell engagers could counter immune escape mechanisms [5].

The highest age‐standardized incidence rate is over 100 per 100,000 men in developed regions such as North America, Western Europe, and Australia; however, much lower rates are seen in Asian countries with rates gradually rising because of more efficient diagnostic techniques and alterations in life style [1, 14]. Age is considered the most important risk factor since the risk of the disease increases exponentially in individuals who have crossed 50 years of age, and African Americans have nearly 1.7 times higher incidence and 2–3 fold higher mortality compared with other racial groups [15]. Family history of the disease increases the risk by 2–3 folds, especially in those who have more than one first degree relative with the disease and with early onset disease [16].

Prostate cancer′s classification and risk stratification take into account histological grading, clinical staging, and serum prostate‐specific antigen (PSA) levels. The Gleason grading system, which has been modified to the five‐grade Grade Group system through the International Society of Urological Pathology (ISUP) in 2014, still forms the basis of histological grading [17]. Clinical staging employs the TNM (tumor, node, metastasis) classification system, whereas the D′Amico classification and the National Comprehensive Cancer Network (NCCN) guidelines classify patients into low, intermediate, or high‐risk depending on their PSA levels, Grade Group, and clinical staging [17]. These systems are important in the choice of management strategy from active surveillance for low‐risk patients to multimodal therapy in the case of high‐risk cases. An understanding of these epidemiological trends, risk factors, and classification systems is crucial for early detection, proper risk stratification, and patient management through proper selection of treatment modality.

## 2. TMB and Immunogenicity in Prostate Cancer

TMB is a principal determinant of tumor immunogenicity and a recognized correlate of checkpoint inhibitor responsiveness across cancer types [18]. Relative to highly immunogenic malignancies such as melanoma or smoking‐associated lung cancer, prostate cancer typically exhibits a markedly lower mutational burden—estimated at roughly 7‐fold to 15‐fold lower than melanoma [19].

This limited immunogenicity is mirrored histologically by sparse infiltration of CD8+ cytotoxic T lymphocytes. Many prostate tumors are accordingly classified as immunologically cold, displaying low expression of interferon‐gamma (IFN‐γ)‐responsive gene signatures, restricted antigen presentation machinery, and inadequate priming of adaptive immunity [20].

Nonetheless, a clinically important minority of prostate cancers demonstrates heightened immunogenicity as a consequence of DNA damage repair pathway defects. Germline and somatic alterations in BRCA1, BRCA2, ATM, checkpoint kinase 2 (CHEK2), partner and localizer of BRCA2 (PALB2), and CDK12 have been identified in a substantial proportion of men with metastatic castration‐resistant prostate cancer (mCRPC) [11, 21]. These alterations promote genomic instability and neoantigen formation, thereby increasing the likelihood of an effective antitumor immune response.

The problem with treating prostate cancer using immunotherapy is because of the very low level of TMB, immunosuppressive myeloid microenvironment and weak T‐cell infiltration, which has resulted in modest rates of response to immune checkpoint inhibitors, vaccines and adoptive cell therapy in prostate cancer patients using monotherapy alone. The authors strongly argue that the key to successful prostate cancer immunotherapy will be based on using combination therapies, using biomarkers for patient selection and practicing personalized medicine in order to make the tumor microenvironment “hot” for selected patients [22].

The microsatellite instability high/mismatch repair deficient (MSI‐H/dMMR) prostate cancers represent a distinct and particularly immunotherapy‐responsive subgroup. A large genomic analysis by Lenis and colleagues [13], sequencing over 3200 tumors from more than 2200 patients, found that MSI‐H/dMMR cancers carry significantly greater TMB, indel burden, and neoantigen load than tumors that are TMB‐high but microsatellite stable, and that this distinction correlated with more durable responses to checkpoint blockade. Complementary work by Abida and colleagues [12] similarly demonstrated that mismatch repair deficiency, although present in only a small fraction of unselected mCRPC cases, identifies patients likely to benefit substantially from PD‐1 inhibition, consistent with tissue‐agnostic regulatory approval of pembrolizumab for MSI‐H/dMMR solid tumors [13, 18].

INF‐γ signaling constitutes a second key determinant of immunotherapy responsiveness. IFN‐*γ* pathway activation enhances antigen presentation, upregulates major histocompatibility complex expression, and promotes T‐cell recruitment; however, chronic or sustained IFN‐*γ* signaling can paradoxically favor immune escape by inducing compensatory PD‐L1 expression and other inhibitory feedback loops [20].

## 3. Tumor Immune Microenvironment (TME) in Prostate Cancer

The TME of prostate cancer is highly immunosuppressive and represents a principal barrier to effective checkpoint blockade [8]. Multiple interacting cellular and molecular programs converge to restrain antitumor immunity and facilitate disease progression.

### 3.1. TAMs

The TAMs, particularly those polarized toward the M2 phenotype, play a central role in prostate cancer progression and therapeutic resistance [23]. Increased infiltration of CD163+ macrophages correlates with neuroendocrine differentiation, enzalutamide resistance, metastatic spread, and inferior clinical outcomes.

Experimental work by Wang and colleagues [24] demonstrated that androgen receptor inhibition paradoxically increases secretion of high‐mobility group box 1 protein (HMGB1) from prostate cancer cells, which recruits and activates TAMs. These macrophages, in turn, secrete IL‐6, which promotes neuroendocrine differentiation and therapy resistance via signal transducer and activator of transcription 3 (STAT3) signaling —establishing a feedback loop that can be therapeutically interrupted with macrophage‐targeted agents.

Recent reports have suggested that clinical response to hormone therapy to a lot of extent is dictated by TAMs [25]. TAMs further contribute to immune evasion through secretion of IL‐10, TGF‐*β*, VEGF, and prostaglandin E2, which collectively inhibit cytotoxic T‐cell function while promoting tumor angiogenesis [3].

### 3.2. Regulatory T Cells and MDSCs

Regulatory T cells suppress antitumor immunity through secretion of IL‐10 and TGF‐*β* and via direct inhibition of effector T‐cell proliferation [8]. Elevated intratumoral Treg infiltration is consistently associated with adverse prognosis in prostate cancer. MDSCs similarly inhibit T‐cell activation through generation of reactive oxygen species (ROS), inducible nitric oxide synthase (iNOS), arginase‐1, and additional immunosuppressive cytokines [3].

Loss of phosphatase and tensin homolog (PTEN) and consequent phosphoinositide 3‐kinase (PI3K) pathway activation strongly promote recruitment of both MDSCs and Tregs within the prostate TME [26]. Notably, Qi and colleagues [26] demonstrated that intermittent, rather than continuous, dosing of a PI3K*α*/*β*/*δ* inhibitor (BAY1082439) reprograms PTEN‐null prostate tumors from an immunosuppressive to an immune‐stimulatory state—increasing interferon pathway activation, *β*2‐microglobulin expression, and C‐X‐C motif chemokine ligand (CXCL10)/C‐C motif chemokine ligand 5 (CCL5) secretion, while preferentially depleting Tregs and expanding tumor‐associated CD8+ T cells. This intermittent dosing strategy restored sensitivity to PD‐1 blockade in preclinical models, illustrating that scheduling, not merely target selection, can determine whether targeted therapy synergizes with or antagonizes checkpoint inhibition.

### 3.3. CAFs and Additional Stromal Contributions

The CAFs further reinforce the immunosuppressive prostate TME by remodeling extracellular matrix architecture, secreting TGF‐*β* and other inhibitory cytokines, and physically restricting T‐cell infiltration into tumor nests [9]. Together with TAMs, Tregs, and MDSCs, CAFs form an interconnected suppressive network that must be disrupted at multiple nodes for checkpoint blockade to achieve durable efficacy.

## 4. Regulation of T Cells and Immune Checkpoint Inhibitors

Prostate cancer linked to immunosuppression is characterized by reduced cytotoxic T‐cell activity, impaired antigen presentation, and increased levels of immunosuppressive cytokines and immune checkpoint molecules [9, 10] T‐cells possess receptors that specifically bind to nonself‐structures, leading to immune system activation. However, activation of a complete immune response also requires additional proteins that act as T‐cell accelerators. In addition, some proteins function as inhibitors or brakes of T‐cells, thereby restraining immune activation, such as the cytotoxic T‐Lymphocyte–associated antigen 4 (CTLA‐4) and PD‐1 PD‐L1) and PD‐1 are crucial in enabling neoplastic cells to evade detection and destruction by the immune system, a process known as immune evasion [10, 27]. Additionally, PD‐L1 (SP263) expression is positively associated with aggressive characteristics in the pathogenesis of prostate cancer [27].

This delicate balance between the accelerators and inhibitors is critical for precise regulation. Based on these findings, a new therapeutic approach was developed for cancer using immune checkpoint inhibitors. These work by blocking the negative regulatory pathways, such as the PD‐1/PD‐L1 pathway, that are used by cancer cells to evade the immune system [28–30]. PD‐L1 is expressed on the surface of cancer cells, and when it interacts with PD‐1 on the surface of immune cells, it sends a signal that inhibits the immune response. By blocking this interaction, immune checkpoint inhibitors can restore the immune response and help the body fight the cancer. The immune checkpoint inhibitors have been shown to be effective in advanced prostate cancer (Figure 1).

**Figure 1:**
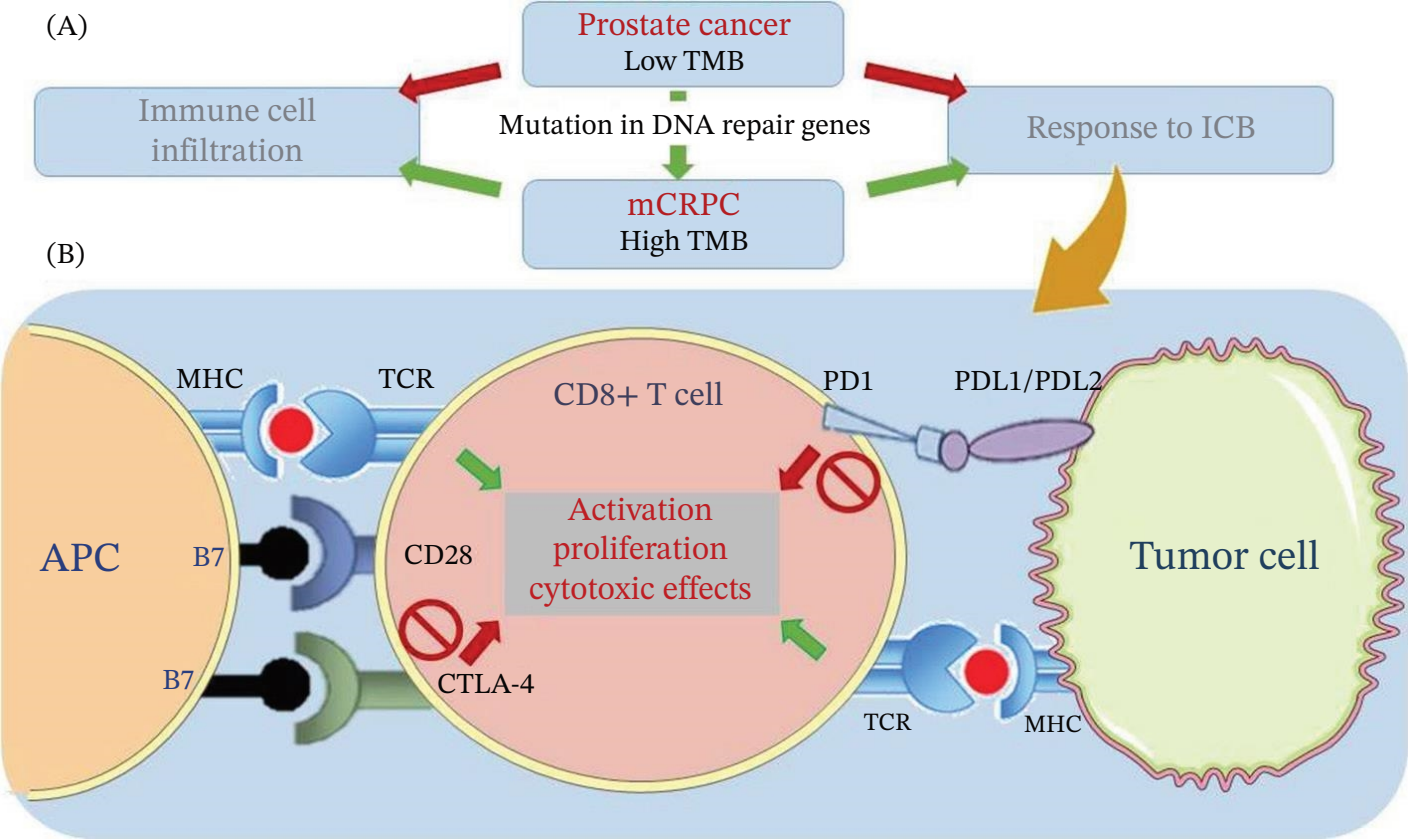
Molecular mechanisms of immune checkpoint inhibitors and inhibition of negative immune regulation in prostate cancer. (A) Prostate cancer has a low level of tumor mutation burden (TMB), which means it has fewer immune cell infiltrations, particularly T cells, in the tumor tissue. This can result in a weak response to immune checkpoint blockade (ICB). However, DNA repair gene mutations, particularly in homologous recombination repair genes have been identified in a significant percentage of patients with metastatic castration‐resistant prostate cancer (mCRPC), which increase the TMB and thereby the responsiveness to ICB. (B) Tumor antigens stimulate T‐cells through interactions between antigen‐presenting cells (APCs) and major histocompatibility complex (MHC) molecules. Complete activation of T‐cells requires additional costimulatory signals. Negative modulators such as CTLA‐4 and programmed cell death protein 1/programmed death‐ligand 1 regulate the immune response. cytotoxic T‐Lymphocyte–associated antigen 4 has a higher affinity for binding to B7 than CD28, leading to the inhibition of T‐cell activity. The PD‐1/PD‐L1 pathway regulates inflammation and prevents damage to normal tissues, but cancer cells may exploit it. Blockers of CTLA‐4 or PD‐1/PD‐L1 can enhance T‐cell activity, thereby augmenting the anti‐tumor response. Green arrows indicate activation; red arrows indicate inhibition.

## 5. Immune Checkpoint Pathways in Prostate Cancer

Immune checkpoints are inhibitory signaling pathways that normally maintain immune homeostasis and prevent autoimmunity. Cancer cells exploit these physiological brakes to evade immune‐mediated destruction [10].

### 5.1. PD‐1/PD‐L1 Axis

PD‐1 is expressed on activated T cells, B cells, and natural killer (NK) cells [30]). Engagement of PD‐1 by its ligands PD‐L1 or PD‐L2 suppresses T‐cell activation, cytokine secretion, and clonal proliferation. Prostate cancer cells and tumor‐infiltrating immune cells express PD‐L1 with considerable heterogeneity across studies, largely reflecting differences in antibody clone, scoring methodology, and tissue handling [27].

A recent analysis by Zhuang and colleagues [27], using the SP263 PD‐L1 assay, found that PD‐L1 positivity correlates with adverse clinicopathological features, including higher Gleason score, elevated proliferative index, neuroendocrine differentiation, and inferior prognosis—supporting a role for PD‐L1 as both a prognostic marker and a rationale for checkpoint blockade in a biomarker‐selected subset of patients.

### 5.2. CTLA‐4 Signaling

CTLA‐4 acts principally during the early phase of T‐cell activation, competing with the costimulatory receptor CD28 for binding to CD80/CD86 on antigen‐presenting cells. Because CTLA‐4 binds these ligands with substantially higher affinity than CD28, its expression effectively dampens T‐cell priming and proliferation [29].

Blockade of CTLA‐4 with ipilimumab can restore T‐cell activity and promote antitumor immunity. However, in prostate cancer specifically, CTLA‐4 inhibition as monotherapy has yielded limited efficacy while producing substantial immune‐related adverse events, a pattern reflected in the negative Phase III trials of ipilimumab in mCRPC [2, 31].

### 5.3. Emerging Checkpoint Targets: TIGIT, LAG‐3, and Adenosine Signaling

Beyond PD‐1/PD‐L1 and CTLA‐4, additional inhibitory pathways contribute to immune evasion in prostate cancer. T‐cell immunoreceptor with immunoglobulin and ITIM domain (TIGIT), an inhibitory receptor expressed on T and NK cells, competes with the activating receptor CD226 for shared ligands and is increasingly implicated in checkpoint inhibitor resistance across solid tumors [3]. Lymphocyte‐activation gene 3 (LAG‐3) similarly restrains T‐cell effector function and is under early clinical investigation in combination with PD‐1 blockade. Extracellular adenosine, generated through CD39/CD73‐mediated adenosine triphosphate (ATP) catabolism within the hypoxic prostate TME, further suppresses T‐cell and NK‐cell function via A2A receptor signaling, representing another rational combination target [9].

## 6. Clinical Trials of Immune Checkpoint Inhibitors in Prostate Cancer

Clinical trials evaluating immune checkpoint inhibitors in prostate cancer have produced heterogeneous and generally modest results, reflecting the immunologically cold nature of the disease in unselected populations [2] Table 1.

**Table 1:** Summary of key clinical trials evaluating immune checkpoint inhibitors in prostate cancer.

Trial	Regimen	Population	Key finding	References
KEYNOTE‐199	Pembrolizumab (monotherapy)	Treatment‐refractory mCRPC (3 cohorts)	Modest ORR (−5% even among PD‐L1‐positive patients)	[32]
CA184‐043/CA184‐095	Ipilimumab (monotherapy)	mCRPC	Did not meet Primary overall survival endpoint, signals of activity in favorable‐prognosis subgroups	[33]
Durvalumab + Olaparib	PD‐L1 inhibitor + PARP inhibitor	mCRPC postenzalutamide/abiraterone	Median rPFS of 16.1months in DDR‐altered patients versus markedly shorter in biomarker‐negative patients	[34]
Pembrolizumab + Cryotherapy	PD‐1 inhibitor + prostatic cryoablation	Newly diagnosed oligometastatic, hormone‐sensitive PCa	Feasible combination activity	[35]
Enzalutamide + Pembrolizumab	AR pathway inhibitor + PD‐1 inhibitor	Biomarker‐unselected mCRPC	Responses reported even without biomarker selection, including occasional deep, durable responses	[36]
INSPIRE	Ipilimumab + Nivolumab (dual checkpoint blockade)	Biomarker‐selected mCRPC	Preselection for DDR deficiency	[37]
NEPTUNES	Checkpoint blockade in molecularly selected patients	Immunogenic molecular signature	Improved response rates with molecular preselection	[38]

### 6.1. Monotherapy Trials

Early trials of PD‐1/PD‐L1 inhibitor monotherapy demonstrated only modest activity in unselected mCRPC populations. The multicohort, open‐label Phase II KEYNOTE‐199 study evaluated pembrolizumab in treatment‐refractory mCRPC across three parallel cohorts and observed objective responses in a limited subset of patients, with more consistent activity among those with PD‐L1‐positive tumors or DNA damage repair alterations [32]. Notably, overall response rates in this and related monotherapy series have remained low—on the order of 5% even among PD‐L1‐positive patients—underscoring that PD‐1 blockade alone is insufficient for most men with mCRPC [32].

Ipilimumab monotherapy has likewise shown limited single‐agent activity. The Phase III CA184‐095 and CA184‐043 trials of ipilimumab in mCRPC did not meet their primary overall survival endpoints, despite signals of activity in prespecified subgroups with more favorable prognostic features [2, 31].

### 6.2. Combination with poly ADP ribose polymerase (PARP) Inhibitors

Combination approaches have emerged as more promising strategies than monotherapy. PARP inhibitors may synergize with checkpoint blockade by increasing genomic instability, neoantigen production, and activation of the cytosolic DNA‐sensing stimulator of interferon genes (STING) pathway [34]. In a Phase II study of durvalumab plus olaparib in men with mCRPC previously treated with enzalutamide and/or abiraterone, Karzai and colleagues [34] reported a median radiographic progression‐free survival of 16.1 months among patients with DNA damage repair alterations, compared with markedly shorter progression‐free survival in biomarker‐negative patients—findings that were later corroborated by biomarker analyses linking baseline MDSC fraction to treatment response. These results support DNA damage repair (DDR) status as a clinically actionable biomarker for PARP inhibitor–checkpoint inhibitor combinations (Figure 2).

**Figure 2:**
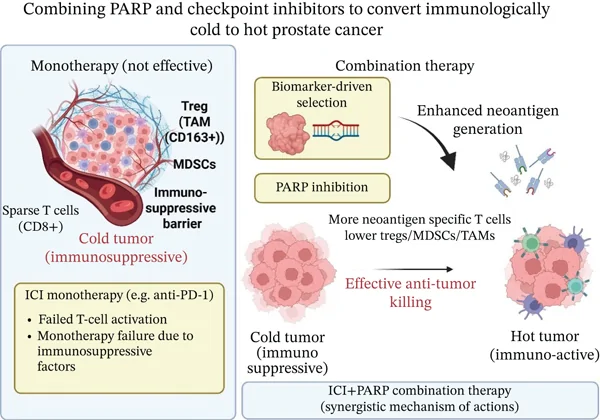
Synergistic mechanism of poly ADP ribose polymerase (PARP) and immune checkpoint inhibition in converting immunologically “cold” prostate tumors.

### 6.3. Combination with Radiotherapy and Cryotherapy

Radiotherapy and cryoablation may potentiate antitumor immunity through induction of immunogenic cell death, release of tumor‐associated antigens, and activation of innate immune sensing pathways [35]. A pilot trial combining pembrolizumab with prostatic cryotherapy in men with newly diagnosed oligometastatic, hormone‐sensitive prostate cancer demonstrated the feasibility and preliminary immunologic activity of this combination, supporting further investigation of local ablative therapy as an immune‐priming strategy [35].

### 6.4. Combination with Androgen Receptor Pathway Inhibitors

Androgen receptor signaling inhibition may enhance antitumor immunity by promoting thymic regeneration, increasing T‐cell infiltration, and modulating macrophage polarization, providing rationale for combining enzalutamide or abiraterone with checkpoint blockade [2]. Early‐phase combination studies pairing enzalutamide with pembrolizumab have reported responses even among biomarker‐unselected patients, including occasional deep and durable responses, although confirmatory randomized data remain limited.

### 6.5. Additional Combination Strategies

Further combinatorial strategies under active investigation include checkpoint inhibitors paired with cancer vaccines, prostate‐specific membrane antigen (PSMA)‐targeted radioligand and immunotherapies, tyrosine kinase inhibitors (TKIs), cytotoxic chemotherapy, and granulocyte‐macrophage colony‐stimulating factor (GM‐CSF)‐based approaches [39]. Dual checkpoint blockade combining CTLA‐4 and PD‐1 inhibition has also been explored in molecularly selected populations; the Phase II INSPIRE trial of ipilimumab plus nivolumab in biomarker‐selected mCRPC and the NEPTUNES trial in patients with an immunogenic molecular signature both suggest that preselection for DDR deficiency, MSI‐H/dMMR status, or other immunogenic features is essential to unlocking meaningful clinical benefit from dual checkpoint blockade.

## 7. Predictive Biomarkers of Response

Given the modest efficacy of checkpoint blockade in unselected prostate cancer populations, identification of predictive biomarkers is essential to appropriately match patients to therapy. Currently, the most clinically validated biomarkers are MSI‐H/dMMR status and TMB, both of which carry tissue‐agnostic regulatory approval for pembrolizumab [13, 18]. DDR gene alterations, particularly biallelic BRCA2 loss and CDK12 biallelic inactivation, represent additional candidate biomarkers, with CDK12‐altered tumors showing a distinct pattern of focal tandem duplications that generates gene fusion neoantigens and increased immune infiltration [11, 40]. PD‐L1 expression, although prognostically informative, has shown inconsistent predictive value for response, likely reflecting assay heterogeneity and the dynamic, inducible nature of PD‐L1 expression [27]. Circulating biomarkers, including baseline MDSC fraction and circulating tumor cell dynamics, have shown early promise as pharmacodynamic and predictive markers in combination trials [34].

## 8. Mechanisms of Resistance

Resistance to checkpoint blockade in prostate cancer arises through both tumor‐intrinsic and microenvironmental mechanisms. Tumor‐intrinsic resistance includes loss of antigen presentation machinery, downregulation of major histocompatibility complex Class I expression, and defects in interferon signaling pathways that blunt the response to immune attack [20]. Microenvironmental resistance mechanisms include persistent recruitment of Tregs and MDSCs, physical exclusion of T cells by dense stromal and CAF‐rich architecture, and compensatory upregulation of alternative inhibitory checkpoints such as TIGIT and LAG‐3 following PD‐1 blockade [3, 9]. PTEN loss and consequent PI3K pathway hyperactivation represent a particularly well‐characterized resistance mechanism, one that can be reversed pharmacologically through intermittent, schedule‐optimized PI3K inhibition [26].

## 9. Future Directions and Emerging Strategies

Converting prostate cancer from an immunologically cold to a hot tumor will likely require rationally designed, multimodal combination strategies rather than any single intervention. Promising avenues include sequencing or combining PARP inhibitors with checkpoint blockade in DDR‐deficient disease, schedule‐optimized PI3K inhibition in PTEN‐null tumors, local ablative therapies (radiotherapy, cryotherapy) to prime systemic immunity ahead of checkpoint blockade, PSMA‐directed therapies to debulk disease and release tumor antigen, and adoptive cellular therapies including CAR T cells and bispecific T‐cell engagers directed against prostate‐specific antigens [26, 34, 39]. Prospective biomarker‐driven trial designs that enrich for DDR‐deficient, MSI‐H/dMMR, or CDK12‐altered tumors will be essential to demonstrate efficacy signals that may otherwise be diluted in unselected populations.

## 10. Conclusions

Prostate cancer remains a biologically heterogeneous and therapeutically challenging malignancy. Although immune checkpoint inhibitors have transformed the treatment landscape for several solid tumors, their efficacy in prostate cancer has thus far been constrained by a profoundly immunosuppressive tumor microenvironment, low TMB, sparse T‐cell infiltration, and multiple adaptive resistance mechanisms [8].

Nevertheless, substantial progress has been made in delineating the molecular and immunological mechanisms underlying immune evasion in prostate cancer. Emerging evidence demonstrates that specific molecular subsets—particularly tumors harboring DDR defects, MSI‐H/dMMR alterations, and CDK12 mutations—exhibit meaningfully enhanced responsiveness to immunotherapy [13].

Current research increasingly supports the view that successful immunotherapy in prostate cancer will require multimodal strategies capable of simultaneously targeting several resistance mechanisms at once (Figure 3). Combination approaches integrating checkpoint blockade with PARP inhibitors, androgen receptor pathway inhibitors, radiotherapy, PSMA‐targeted agents, PI3K inhibitors, cancer vaccines, and adoptive cellular therapies hold considerable promise for improving antitumor immune responses in this historically immunotherapy‐resistant disease [26, 34].

**Figure 3:**
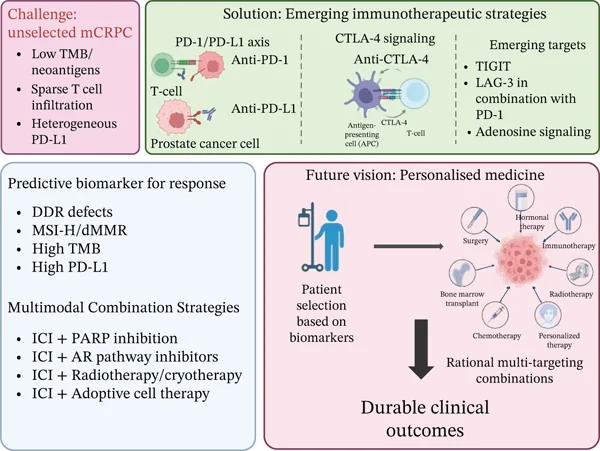
Current challenges, biomarker‐driven strategies, and future multimodal approaches for immunotherapy in metastatic castration‐resistant prostate cancer (mCRPC).

## Author Contributions

Conceptualization: S.D., P.S., and S.R. Writing—original draft: S.D., P.S., U.C., and S.R. Writing—review and editing: I.M.R., J.C.K., and M.K.P.S.

## Funding

No funding was received for this manuscript.

## Disclosure

All authors have read and approved the manuscript. All content was critically reviewed and approved by the authors, who take full responsibility for the final manuscript.

## Ethics Statement

The authors have nothing to report.

## Consent

The authors have nothing to report.

## Conflicts of Interest

The authors declare no conflict of interest.

## Data Availability

Data sharing not applicable to this article as no datasets were generated or analysed during the current study.

## References

[1] Sung H. , Ferlay J. , Siegel R. L. , Laversanne M. , Soerjomataram I. , Jemal A. , and Bray F. , Global Cancer Statistics 2020: GLOBOCAN Estimates of Incidence and Mortality Worldwide for 36 Cancers in 185 Countries, CA: A Cancer Journal for Clinicians. (2021) 71, no. 3, 209–249, 10.3322/caac.21660, 33538338. 33538338

[2] Venkatachalam S. , McFarland T. R. , Agarwal N. , and Swami U. , Immune Checkpoint Inhibitors in Prostate Cancer, Cancers. (2021) 13, no. 9, 10.3390/cancers13092187, 34063238. PMC8125096 34063238

[3] Hu S.-Q. , Dong J.-H. , and Chang Y.-F. , Progress in the Immune Microenvironment of Prostate Cancer and New Immunotherapy Strategies, Cancer Advances. (2025) 8, e25015–e25030, 10.53388/2025825015.

[4] Lu H. , Teng Z. , Wang J. , and Zhang W. , Prostate Cancer Immunotherapy-Based Strategies: An Updated Review Emphasizing Immune Checkpoint Inhibitors, Frontiers in Immunology. (2025) 16, 10.3389/fimmu.2025.1583363, 40607417. PMC12213564 40607417

[5] Wang N. , Wang C. , Cui S. , Zhao K. , Zhang J. , Zhang L. , Jia Y. , and Kong F. , Recent Advances and Future Prospects of Immunotherapeutic Approaches in Prostate Cancer, Journal of Translational Medicine. (2026) 24, no. 1, 10.1186/s12967-026-07720-2, 41546087. PMC12892485 41546087

[6] Ellis P. M. , Vella E. T. , and Ung Y. C. , Immune Checkpoint Inhibitors for Patients With Advanced Non–Small-Cell Lung Cancer: A Systematic Review, Clinical Lung Cancer. (2017) 18, no. 5, 444–459, 10.1016/j.cllc.2017.02.001. 28416123

[7] Wolchok J. D. , Kluger H. , Callahan M. K. , Postow M. A. , Rizvi N. A. , Lesokhin A. M. , Segal N. H. , Ariyan C. E. , Gordon R.-A. , Reed K. , Burke M. M. , Caldwell A. , Kronenberg S. A. , Agunwamba B. U. , Zhang X. , Lowy I. , Inzunza H. D. , Feely W. , Horak C. E. , Hong Q. , Korman A. J. , Wigginton J. M. , Gupta A. , and Sznol M. , Nivolumab Plus Ipilimumab in Advanced Melanoma, New England Journal of Medicine. (2013) 369, no. 2, 122–133, 10.1056/nejmoa1302369, 23724867. PMC5698004 23724867

[8] Stultz J. and Fong L. , How to Turn up the Heat on the Cold Immune Microenvironment of Metastatic Prostate Cancer, Prostate Cancer and Prostatic Diseases. (2021) 24, no. 3, 697–717, 10.1038/s41391-021-00340-5, 33820953. PMC8384622 33820953

[9] Novysedlak R. , Guney M. , Al Khouri M. , Bartolini R. , Koumbas Foley L. , Benesova I. , Ozaniak A. , Novak V. , Vesely S. , Pacas P. , Buchler T. , and Ozaniak Strizova Z. , The Immune Microenvironment in Prostate Cancer: A Comprehensive Review, Oncology. (2025) 103, no. 6, 521–545, 10.1159/000541881, 39380471. PMC12140600 39380471

[10] Barach Y. S. , Lee J. S. , and Zang X. , T Cell Coinhibition in Prostate Cancer: New Immune Evasion Pathways and Emerging Therapeutics, Trends in Molecular Medicine. (2011) 17, no. 1, 47–55, 10.1016/j.molmed.2010.09.006. PMC3039708 20971039

[11] Ryan M. J. and Bose R. , Genomic Alteration Burden in Advanced Prostate Cancer and Therapeutic Implications, Frontiers in Oncology. (2019) 9, 10.3389/fonc.2019.01287, 31824860. PMC6882947 31824860

[12] Abida W. , Cheng M. L. , Armenia J. , Middha S. , Autio K. A. , Vargas H. A. , Rathkopf D. , Morris M. J. , Danila D. C. , Slovin S. F. , Carbone E. , Barnett E. S. , Hullings M. , Hechtman J. F. , Zehir A. , Shia J. , Jonsson P. , Stadler Z. K. , Srinivasan P. , Laudone V. P. , Reuter V. , Wolchok J. D. , Socci N. D. , Taylor B. S. , Berger M. F. , Kantoff P. W. , Sawyers C. L. , Schultz N. , Solit D. B. , Gopalan A. , and Scher H. I. , Analysis of the Prevalence of Microsatellite Instability in Prostate Cancer and Response to Immune Checkpoint Blockade, JAMA Oncology. (2019) 5, no. 4, 471–478, 10.1001/jamaoncol.2018.5801, 30589920. PMC6459218 30589920

[13] Lenis A. T. , Ravichandran V. , Brown S. , Alam S. M. , Katims A. , Truong H. , Reisz P. A. , Vasselman S. , Nweji B. , Autio K. A. , Morris M. J. , Slovin S. F. , Rathkopf D. , Danila D. , Woo S. , Vargas H. A. , Laudone V. P. , Ehdaie B. , Reuter V. , Arcila M. , Berger M. F. , Viale A. , Scher H. I. , Schultz N. , Gopalan A. , Donoghue M. T. A. , Ostrovnaya I. , Stopsack K. H. , Solit D. B. , and Abida W. , Microsatellite Instability, Tumor Mutational Burden, and Response to Immune Checkpoint Blockade in Patients With Prostate Cancer, Clinical Cancer Research. (2024) 30, no. 17, 3894–3903, 10.1158/1078-0432.ccr-23-3403, 38949888. PMC11371520 38949888

[14] Bray F. , Ferlay J. , Soerjomataram I. , Siegel R. L. , Torre L. A. , and Jemal A. , Global Cancer Statistics 2018: GLOBOCAN Estimates of Incidence and Mortality Worldwide for 36 Cancers in 185 Countries, CA: A Cancer Journal for Clinicians. (2018) 68, no. 6, 394–424, 10.3322/caac.21492, 30207593. 30207593

[15] Siegel R. L. , Miller K. D. , Fuchs H. E. , and Jemal A. , Cancer Statistics, 2021, CA: A Cancer Journal for Clinicians. (2021) 71, no. 1, 7–33, 10.3322/caac.21654. 33433946

[16] Pritchard C. C. , Mateo J. , Walsh M. F. , De Sarkar N. , Abida W. , Beltran H. , Garofalo A. , Gulati R. , Carreira S. , Eeles R. , Elemento O. , Rubin M. A. , Robinson D. , Lonigro R. , Hussain M. , Chinnaiyan A. , Vinson J. , Filipenko J. , Garraway L. , Taplin M.-E. , AlDubayan S. , Han G. C. , Beightol M. , Morrissey C. , Nghiem B. , Cheng H. H. , Montgomery B. , Walsh T. , Casadei S. , Berger M. , Zhang L. , Zehir A. , Vijai J. , Scher H. I. , Sawyers C. , Schultz N. , Kantoff P. W. , Solit D. , Robson M. , Van Allen E. M. , Offit K. , de Bono J. , and Nelson P. S. , Inherited DNA-Repair Gene Mutations in Men With Metastatic Prostate Cancer, New England Journal of Medicine. (2016) 375, no. 5, 443–453, 10.1056/nejmoa1603144, 27433846. PMC4986616 27433846

[17] Epstein J. I. , Egevad L. , Amin M. B. , Delahunt B. , Srigley J. R. , Humphrey P. A. , and Grading Committee , The 2014 International Society of Urological Pathology (ISUP) Consensus Conference on Gleason Grading of Prostatic Carcinoma, American Journal of Surgical Pathology. (2016) 40, no. 2, 244–252, 10.1097/pas.0000000000000530, 26492179. 26492179

[18] Marabelle A. , Fakih M. , Lopez J. , Shah M. , Shapira-Frommer R. , Nakagawa K. , Chung H. C. , Kindler H. L. , Lopez-Martin J. A. , Miller W. H. , Italiano A. , Kao S. , Piha-Paul S. A. , Delord J. P. , McWilliams R. R. , Fabrizio D. A. , Aurora-Garg D. , Xu L. , Jin F. , Norwood K. , and Bang Y. J. , Association of Tumour Mutational Burden With Outcomes in Patients With Advanced Solid Tumours Treated With Pembrolizumab: Prospective Biomarker Analysis of the Multicohort, Open-Label, Phase 2 KEYNOTE-158 Study, Lancet Oncology. (2020) 21, no. 10, 1353–1365, 10.1016/s1470-2045(20)30445-9, 32919526. 32919526

[19] Berger M. F. , Lawrence M. S. , Demichelis F. , Drier Y. , Cibulskis K. , Sivachenko A. Y. , Sboner A. , Esgueva R. , Pflueger D. , Sougnez C. , Onofrio R. , Carter S. L. , Park K. , Habegger L. , Ambrogio L. , Fennell T. , Parkin M. , Saksena G. , Voet D. , Ramos A. H. , Pugh T. J. , Wilkinson J. , Fisher S. , Winckler W. , Mahan S. , Ardlie K. , Baldwin J. , Simons J. W. , Kitabayashi N. , MacDonald T. Y. , Kantoff P. W. , Chin L. , Gabriel S. B. , Gerstein M. B. , Golub T. R. , Meyerson M. , Tewari A. , Lander E. S. , Getz G. , Rubin M. A. , and Garraway L. A. , The Genomic Complexity of Primary Human Prostate Cancer, Nature. (2011) 470, no. 7333, 214–220, 10.1038/nature09744, 21307934. PMC3075885 21307934

[20] Reva B. A. , Omelchenko T. , Nair S. S. , and Tewari A. K. , Immune Escape in Prostate Cancer, Urologic Clinics. (2020) 47, no. 4, e9–e16, 10.1016/j.ucl.2020.10.004. 33446324

[21] Robinson L. , Gallos I. D. , Conner S. J. , Rajkhowa M. , Miller D. , Lewis S. , Kirkman-Brown J. , and Coomarasamy A. , The Effect of Sperm DNA Fragmentation on Miscarriage Rates: A Systematic Review and Meta-Analysis, Human Reproduction. (2012) 27, no. 10, 2908–2917, 10.1093/humrep/des261, 22791753. 22791753

[22] Flores-Islas A. K. , Rico-Fuentes C. , Sierra-Díaz E. , García-Chagollán M. , Pereira-Suárez A. L. , Zepeda-Nuño J. S. , Moreno-Ortiz J. M. , and Ramírez-De-Arellano A. , Immunotherapeutic Strategies for Prostate Cancer: A Comprehensive Review, Cancers. (2026) 18, no. 2, 10.3390/cancers18020255, 41595174. PMC12839406 41595174

[23] Wang X. and Li Y. , The Disruption of Hematopoiesis in Tumor Progression, Blood Science. (2019) 1, no. 1, 88–91, 10.1097/bs9.0000000000000001, 35402791. PMC8975043 35402791

[24] Wang C. , Peng G. , Huang H. , Liu F. , Kong D. P. , Dong K. Q. , et al., Sun Y. H. , Blocking the Feedback Loop Between Neuroendocrine Differentiation and Macrophages Improves the Therapeutic Effects of Enzalutamide (MDV3100) on Prostate Cancer, Clinical Cancer Research. (2018) 24, no. 3, 708–723. 10.1158/1078-0432.CCR-17-2446 29191973

[25] Castellaro A. M. , Rodriguez-Baili M. C. , Di Tada C. E. , and Gil G. A. , Tumor-Associated Macrophages Induce Endocrine Therapy Resistance in ER+ Breast Cancer Cells, Cancers. (2019) 11, no. 2, 10.3390/cancers11020189. PMC6406935 30736340

[26] Qi Z. , Xu Z. , Zhang L. , Zou Y. , Li J. , Yan W. , Li C. , Liu N. , and Wu H. , Overcoming Resistance to Immune Checkpoint Therapy in PTEN-Null Prostate Cancer by Intermittent Anti-PI3K*α*/*β*/*δ* Treatment, Nature Communications. (2022) 13, no. 1, 10.1038/s41467-021-27833-0, 35013322. PMC8748754 35013322

[27] Zhuang X. , Li H. , You Z. , Cheng H. , Guo G. , Chen X. , and Huang H. , PD-L1 (SP263) Expression Correlates With Pathological Aggressive Parameters in Prostate Cancer, Annals of Diagnostic Pathology. (2025) 78, 152481, 10.1016/j.anndiagpath.2025.152481, 40273724. 40273724

[28] Chowdhury P. , Chamoto K. , and Honjo T. , Combination Therapy Strategies for Improving PD-1 Blockade Efficacy: A New Era in Cancer Immunotherapy, Journal of Internal Medicine. (2018) 283, no. 2, 110–120, 10.1111/joim.12708, 29071761. 29071761

[29] Hodi F. S. , Mihm M. C. , Soiffer R. J. , Haluska F. G. , Butler M. , Seiden M. V. , Davis T. , Henry-Spires R. , Mac Rae S. , and Willman A. , Biologic Activity of Cytotoxic T Lymphocyte-Associated Antigen 4 Antibody Blockade in Previously Vaccinated Metastatic Melanoma and Ovarian Carcinoma Patients, Proceedings of the National Academy of Sciences. (2003) 100, no. 8, 4712–4717, 10.1073/pnas.0830997100, 12682289. PMC153621 12682289

[30] Iwai Y. , Terawaki S. , and Honjo T. , PD-1 Blockade Inhibits Hematogenous Spread of Poorly Immunogenic Tumor Cells by Enhanced Recruitment of Effector T Cells, International Immunology. (2005) 17, no. 2, 133–144, 10.1093/intimm/dxh194, 15611321. 15611321

[31] Kwon Y. , Song W. , Droujinine I. A. , Hu Y. , Asara J. M. , and Perrimon N. , Systemic Organ Wasting Induced by Localized Expression of the Secreted Insulin/IGF Antagonist ImpL2, Developmental Cell. (2015) 33, no. 1, 36–46, 10.1016/j.devcel.2015.02.012, 25850671. PMC4437243 25850671

[32] Antonarakis E. S. , Piulats J. M. , Gross-Goupil M. , Goh J. , Ojamaa K. , Hoimes C. J. , Vaishampayan U. , Berger R. , Sezer A. , Alanko T. , De Wit R. , Li C. , Omlin A. , Procopio G. , Fukasawa S. , Tabata K.-I. , Park S. H. , Feyerabend S. , Drake C. G. , Wu H. , Qiu P. , Kim J. , Poehlein C. , and De Bono J. S. , Pembrolizumab for Treatment-Refractory Metastatic Castration-Resistant Prostate Cancer: Multicohort, Open-Label Phase II KEYNOTE-199 Study, Journal of Clinical Oncology. (2020) 38, no. 5, 395–405, 10.1200/jco.19.01638, 31774688. PMC7186583 31774688

[33] Beer T. M. , Kwon E. D. , Drake C. G. , Fizazi K. , Logothetis C. , Gravis G. , Ganju V. , Polikoff J. , Saad F. , Humanski P. , Piulats J. M. , Gonzalez Mella P. , Ng S. S. , Jaeger D. , Parnis F. X. , Franke F. A. , Puente J. , Carvajal R. , Sengeløv L. , McHenry M. B. , Varma A. , van den Eertwegh A. J. , and Gerritsen W. , Randomized, Double-Blind, Phase III Trial of Ipilimumab Versus Placebo in Asymptomatic or Minimally Symptomatic Patients With Metastatic Chemotherapy-Naive Castration-Resistant Prostate Cancer, Journal of Clinical Oncology. (2017) 35, no. 1, 40–47, 10.1200/jco.2016.69.1584, 28034081. 28034081

[34] Karzai F. , Vander Weele D. , Madan R. A. , Owens H. , Cordes L. M. , Hankin A. , Couvillon A. , Nichols E. , Bilusic M. , Beshiri M. L. , Kelly K. , Krishnasamy V. , Lee S. , Lee M. J. , Yuno A. , Trepel J. B. , Merino M. J. , Dittamore R. , Marté J. , Donahue R. N. , Schlom J. , Killian K. J. , Meltzer P. S. , Steinberg S. M. , Gulley J. L. , Lee J.-M. , and Dahut W. L. , Activity of Durvalumab Plus Olaparib in Metastatic Castration-Resistant Prostate Cancer in Men With and Without DNA Damage Repair Mutations, Journal for Immunotherapy of Cancer. (2018) 6, no. 1, 10.1186/s40425-018-0463-2, 30514390. PMC6280368 30514390

[35] Ross A. E. , Hurley P. J. , Tran P. T. , Rowe S. P. , Benzon B. , Neal T. O. , Chapman C. , Harb R. , Milman Y. , Trock B. J. , Drake C. G. , and Antonarakis E. S. , A Pilot Trial of Pembrolizumab Plus Prostatic Cryotherapy for Men With Newly Diagnosed Oligometastatic Hormone-Sensitive Prostate Cancer, Prostate Cancer and Prostatic Diseases. (2020) 23, no. 1, 184–193, 10.1038/s41391-019-0176-8, 31611635. PMC7031012 31611635

[36] Graff J. N. , Alumkal J. J. , Drake C. G. , Thomas G. V. , Redmond W. L. , Farhad M. , Cetnar J. P. , Ey F. S. , Bergan R. C. , Slottke R. , and Beer T. M. , Early Evidence of Anti-PD-1 Activity in Enzalutamide-Resistant Prostate Cancer, Oncotarget. (2016) 7, no. 33, 52810–52817, 10.18632/oncotarget.10547, 27429197. PMC5288150 27429197

[37] Van Wilpe S. , Kloots I. S. H. , Slootbeek P. H. J. , Den Brok M. , Westdorp H. , Franken M. D. , Coskunturk M. , Osinga T. , Bloemendal H. , Adema G. , Smeenk R. J. , Nagarajah J. , Van Ipenburg J. , Kroeze L. I. , Ligtenberg M. J. L. , Schalken J. , Gerritsen W. R. , and Mehra N. , Ipilimumab With Nivolumab in Molecularly Selected Patients With Castration-Resistant Prostate Cancer: Primary Analysis of the Phase II INSPIRE Trial, Annals of Oncology. (2024) 35, no. 12, 1126–1137, 10.1016/j.annonc.2024.09.004, 39293514. 39293514

[38] Leone G. , Wong Y. N. S. , Jones R. J. , Sankey P. , Josephs D. H. , Crabb S. J. , Harris L. , Zarkar A. , Protheroe A. , Vasudev N. , Rashid M. , Lopes A. , Tasnim A. , Ensell L. , Hartley J. , Jayaram A. , Kularatne B. , Kayani M. , Pritchard C. C. , Konnick E. Q. , Freeman A. , Haider A. , Linares J. , Attard G. , Quezada S. A. , Swanton C. , and Linch M. D. , Nivolumab and Ipilimumab for Metastatic Castration-Resistant Prostate Cancer With an Immunogenic Signature: The Multicenter, Two-Cohort, Phase II NEPTUNES Study, Journal of Clinical Oncology. (2025) 43, no. 28, 3070–3080, 10.1200/jco-24-02637, 40720716. 40720716

[39] Donahue R. N. , Lepone L. M. , Grenga I. , Jochems C. , Fantini M. , Madan R. A. , Heery C. R. , Gulley J. L. , and Schlom J. , Analyses of the Peripheral Immunome Following Multiple Administrations of Avelumab, a Human IgG1 anti-PD-L1 Monoclonal Antibody, Journal for Immunotherapy of Cancer. (2017) 5, no. 1, 10.1186/s40425-017-0220-y, 28239472. PMC5320726 28239472

[40] Wu K. , El Zowalaty A. E. , Sayin V. I. , and Papagiannakopoulos T. , The Pleiotropic Functions of Reactive Oxygen Species in Cancer, Nature Cancer. (2024) 5, no. 3, 384–399, 10.1038/s43018-024-00738-9. 38531982

